# The neuronal ceroid lipofuscinosis type 2 – associated variants: An analysis of alterations in the *TPP1* gene and genotype–phenotype correlation in Ukraine

**DOI:** 10.1002/jmd2.12423

**Published:** 2024-05-14

**Authors:** Nataliia Olkhovych, Nataliia Pichkur, Nataliia Mytsyk, Rodolfo Tonin, Svitlana Kormoz, Iryna Hregul, Nataliia Samonenko, Tetiana Shklyarskaya, Volodymyr Olkhovych, Olexandr Buryak, Amelia Morrone, Nataliia Gorovenko

**Affiliations:** ^1^ Department of Genetic Diagnostics National Scientific Center, Institute of Cardiology, Clinical and Regenerative Medicine M.D. Strazheska, National Academy of Medical Sciences of Ukraine Kyiv Ukraine; ^2^ Laboratory of Medical Genetics National Children's Hospital OHMATDYT, Ministry of Health of Ukraine Kyiv Ukraine; ^3^ Laboratory of Molecular Biology of Neurometabolic Diseases, Neuroscience Department Meyer Children's Hospital (AOU Meyer – IRCCS) Firenze Italy; ^4^ Department of Medical and Laboratory Genetics National University of Health named after P.L.Shupika Kyiv Ukraine; ^5^ Department of Neurosciences, Psychology, Drug Research and Child Health University of Florence Firenze Italy

**Keywords:** genotype–phenotype correlation, neuronal ceroid lipofuscinosis type 2, TPP1 enzyme deficiency, *TPP1 g*ene

## Abstract

The neuronal ceroid lipofuscinosis type 2 (CLN2) is a heterogeneous group of neurodegenerative lysosomal storage disorders caused by autosomal recessive inheritance of two pathogenic variants in trans in the *TPP1* gene. Classical late‐infantile CLN2 disease has a very well‐defined natural history. However, a small number of patients with TPP1 enzyme deficiency present a later onset or protracted disease course within this group there are phenotypic variants. Our work aimed to identify pathological variants in the *TPP1* gene that conditioned the development of CLN2 disease in Ukrainian patients, to compare these variants with those found in patients from other European and non‐European regions, and to make genotype–phenotype associations for this disease. The phenotypes and genotypes of the 48 CLN2‐affected individuals belonging to 43 families were profiled through clinical data collection, enzyme analysis, and genotyping. In most patients, genotype and phenotype correlation are in keeping with the data of previous studies. The clinical signs of the disease in patients with new, previously undescribed variants, allowed us to augment existing data about genotype–phenotype correlations for CLN2 disease. The combination of genotype and clinical form of the disease demonstrated that predicting the type and clinical course of the disease based on genotype is very complicated. The data we obtained supplements existing information on genotype–phenotypic correlations in this rare disease, which, in turn, lays the foundation for a personalized approach to the management of this disease.

**Synopsis**

The phenotypes and genotypes of the 48 CLN2‐affected individuals demonstrated that predicting the type and clinical course of the disease based on genotype is very complicated.

## 1 INTRODUCTION

The neuronal ceroid lipofuscinoses (CLNs) are a heterogeneous group of neurodegenerative lysosomal storage disorders characterized by accumulating neuronal and extraneuronal ceroid lipopigments.^1^ Classical late‐infantile neuronal ceroid lipofuscinosis (CLN2; OMIM ID: 204500) is the result of tripeptidyl‐peptidase 1 (TPP1; EC 3.4.14.9) deficiency, caused by autosomal recessive inheritance of two pathogenic variants in trans in the *TPP1* gene located on chromosome 11p15.4 (Gene ID: 1200).^2^, ^3^, ^4^

CLN2 disease classically presents with seizures onset at 2–4 years of age, preceded by delayed language development and followed by rapidly progressing dementia, psychomotor decline (loss of the ability to walk and talk), epilepsy, blindness, and death, typically between 6 years of age and the early teenage years.^5^, ^6^ Classical late‐infantile CLN2 disease has a very well‐defined natural history. However, a small number of patients with TPP1 enzyme deficiency present a later onset or protracted disease course within this group there are phenotypic variants.^7^

Data on childhood CLN gathered from clinical and molecular studies show a worldwide distribution.^8^ Some studies indicate a higher‐than‐expected incidence in specific geographical regions^9^, ^10^ and may shed light on the origin and distribution of pathological variants. The influence of the type of genetic variant – null variant that leads to the production of a nonfunctional enzyme or no enzyme at all, or residual variant that produces a protein with some function – on the course of the disease has been described for the vast majority of hereditary metabolic diseases, including for CLN2.^11^ That is why the assessment of genotype–phenotype correlation in patients of a certain ethnic origin is so important. All these factors determine the formation of the most effective strategy for the diagnosis and management of this disease in a certain population. Early diagnosis has become increasingly important since the approval of intracerebroventricular enzyme replacement therapy which has been shown to slow the rapid decline in motor and language function in patients.^12^

Our work aimed to identify pathological variants in the *TPP1* gene that conditioned the development of CLN2 disease in Ukrainian patients, to compare these variants with those found in patients from other European and non‐European regions and to make genotype–phenotype associations for this disease.

## 2 PATIENTS AND METHODS

We profiled the phenotypes and genotypes of 48 CLN2 individuals from 43 nonconsanguineous Ukrainian families. The gender ratio (m/f) was 1:1,4. In five of the families, there were two affected siblings. Controls for enzyme and molecular studies were obtained from volunteer donors from a similar ethnic population. Diagnosis of CLN was based on clinical evaluation, neurophysiological investigations (EEG, multiple‐evoked potentials), and neuroradiological findings. All biochemical assays (TPP1 enzyme deficiency) and most molecular analyses (*TPP1* gene variants) were conducted by the National Children's Hospital OHMATDYT. In five patients (7.1, 7.2, 35.1, 37.1, and 38.1), the molecular analysis was conducted by commercial laboratories. In three families (6.1, 16.1, 16.2, and 10.1), molecular analysis was conducted by the Laboratory of Molecular Biology of Neurometabolic Diseases, Meyer Children's Hospital, Florence, Italy. Clinical data were obtained from a medical chart review. DNA was extracted from peripheral blood lymphocytes for molecular genetic testing using a commercial kit NucleoSpin®Blood (Macherey‐Nagel, Duren, Germany). For sequence analysis, primers were designed for polymerase chain reaction (PCR) amplification of all exons and flanking intron sequences of the *TPP1* gene (GenBank ID: AF017456.1, with nucleotide 1 counted as the first nucleotide of the translation initiation codon). Mutation data stated concerning transcript NM_000391.3. Primer sequences are available from the authors upon request. PCR reactions were performed using 10–20 ng of genomic DNA in a volume of 20 μL containing 10 pM of forward and reverse primer, and 10 μL DreamTaq™PCR Master Mix (2x) (Thermoscientific, Vilnius, Lithuania). PCR conditions were as follows: 8 min at 94°C, followed by 35 cycles at 95°C for 30 s, annealing at 58°C for 30 s, and extension at 72°C for 40 s, 1 cycle at 72°C for 7 min. PCR products were purified using a commercial kit NucleoSpin® Gel and PCR Clean‐up (Macherey‐Nagel, Duren, Germany) and sequenced using BigDye Terminator v.3.1 Cycle Sequencing Kit and an ABI 3100 DNA Analyzer (Applied Biosystems). Sequences were analyzed with Sequencing Analysis software 7 (Applied Biosystems). The mutation nomenclature used in this update follows the guidelines indicated by the Human Genome Variation Society.^13^

The variants were identified by comparing bidirectional sequence data against normal control sequences and were confirmed by independent reamplification and bidirectional sequencing from the patients' original DNA. New variants were presumed pathogenic through analysis of cosegregation of the change in the parents. The electronic databases of known mutations – dbSNP, ClinVar, HGMD – were used to characterize the mutations. Various web tools were used for bioinformatic analysis of the new missense variants. The analysis of sequencing results was made using the programs Chromas and BLAST (http://www.ncbi.nlm.nih.gov/blast). The analysis of the pathogenicity of new mutations was made using programs PolyPhen2 and Provean (http://genetics.bwh. harvard.edu/pph2/, http://provean.jcvi.org/index. php), SIFT (https://sift.bii.a-star.edu.sg/), MutationTaster (https://www.mutationtaster.org/).

The type and location of specific variants were compared to the disease phenotypes defined in the CLNet clinical database to establish their severity and identify possible population‐specific variants. In addition, we correlated clinical diagnoses in Ukrainian patients with the frequency and severity of individual mutations. A result was regarded as significant if *p* < 0.05. The Ethical Committee of the Institute of Genetic and Regenerative Medicine gave ethical approval. Informed consent to participate was obtained from parents or guardians of affected individuals.

## 3 RESULTS

The phenotypes and genotypes of the 48 CLN2‐affected individuals belonging to 43 families were profiled through clinical data collection, enzyme analysis, and genotyping (Table 1). All individuals had both alleles identified. In family cases, both siblings had the same genotype, so they were taken as one case when frequency calculating. So, in total, 86 alleles were used for frequency calculation.

**TABLE 1:** The phenotypes and genotypes of the CLN2 affected individuals from Ukraine.

Patients ID	Gender	Age of manifestation, year	Age of diagnosis, year	First symptoms	TPP1 activity, nmol/h/mg protein (reference range 179–681)	Alterations in *TPP1* gene	
						1 allele	2 allele
*Classical late infantile CLN2*							
1.1	f	2,5	4	Seizure	0	p.Arg208*	p.Tyr406*
2.1	f	3	4	Seizure	7,5	p.Arg208*	p.Leu560Thrfs*47
3.1	m	3	4	Seizure	17	p.Arg208*	p.Arg208*
4.1	f	3	4	Seizure	26	p.Leu560Thrfs*47	p.Leu560Thrfs*47
5.1	f	3	4	Seizure	50	p.Ser475Leu	p.Ser475Leu
6.1	m	3,5	5	Seizure	59,4	p.Q75=	p.Q75=
7.1	m	4	5	Seizure	17,5	p.Arg208*	c.509‐1G > C
7.2	f	2	2	Seizure	34	p.Arg208*	c.509‐1G > C
8.1^a^	m	1	1	Seizure	31	p.Arg208*	p.Arg208*
9.1	m	3,5	5	Unsteady gait	52	p.Arg208*	p.Arg208*
11.1	m	3	4	Seizure	6	p.Arg208*	c.1436_1437del CA
12.1	f	4	5	Seizure	39	p.Arg208*	p.Leu560Thrfs*47
15.1	m	2,5	4	Seizure	86	p.Arg208*	p.Arg208*
16.1	m	3	5	Seizure	38	p.Arg208*	p.Q75=
16.2	f	2	3	Seizure	88	p.Arg208*	p.Q75=
17.1	f	3,5	5	Seizure	0	p.Arg208*	p.Arg127*
19.1	f	3,5	5	Seizure	22	p.Arg208*	p.Arg208*
20.1	f	4	5	Seizure	0	p.Arg208*	p.Arg208*
21.1	f	3	4	Seizure	0	p.Arg208*	p.Arg208*
22.1	f	3	4	Seizure	6,5	p.Arg208*	p.Arg208*
23.1	m	3,5	5	Seizure	8,7	p.Arg208*	p.Gln278Pro
24.1	m	4	5	Seizure	13	p.Leu560Thrfs*47	c.509‐1G > C
25.1	m	3,5	4	Seizure	0	p.Arg208*	c.509‐1G > C
25.2	f	4	6	Seizure	12	p.Arg208*	c.509‐1G > C
26.1	f	3	5	Seizure	40	p.Arg208*	p.Arg208*
27.1	f	2	3	Seizure	0	p.Arg208*	p.Arg208*
28.1	f	4	6	Seizure	0	p.Arg208*	p.Arg208*
28.2	f	3	3	Seizure	0	p.Arg208*	p.Arg208*
29.1	f	2	4	Seizure	48	p.Ser475Leu	p.Ser475Leu
30.1	f	3,5	5	Seizure	30,7	p.Arg208*	p.Arg208*
31.1	m	3	4	Seizure	17	p.Arg208*	p.Arg208*
32.1	m	3,9	4	Seizure	0	p.Arg208*	p.Arg208*
33.1	f	2,5	4	Seizure	0	p.Arg208*	p.Leu560Thrfs*47
34.1	m	3,11	5	Unsteady gait	34	p.Arg208*	p.Arg208*
35.1	f	2,5	4	Seizure	37	p.Arg208*	p.Tyr406*
36.1	f	2,8	3	Seizure	0	p.Arg208*	p.Arg208*
38.1	f	3	4	Seizure	0	p.Arg208*	p.Arg208*
39.1	m	2,5	3	Unsteady gait	0	p.Arg208*	p.Arg208*
40.1	f	3	3	Seizure	45	p.Arg208*	p.Arg208*
41.1	f	2	3	Seizure	33	p.Arg208*	p.Arg208*
42.1	m	3	4	Seizure	8,2	p.Arg208*	p.Leu560Thrfs*47
**Mean ± SD**		**3,0 ± 0,1**	**4,1 ± 0,2**		**22,1 ± 3,7**		
*Late‐onset CLN2*							
10.1	f	7	8	Seizure	16	p.Arg208*	p.Q75=
13.1	m	6	11	Speech delay	0	p.Arg208*	p.Met281Ile
14.1	m	7	12	Speech delay	27	p.Arg208*	p.Leu560Thrfs*47
18.1	f	8	11	Seizure	62	p.Arg208*	p.Arg208*
18.2	m	7	7	Unsteady gait	38	p.Arg208*	p.Arg208*
37.1	m	6	9	Unsteady gait	59	p.Arg208*	p.Tyr449Asn
43.1	f	7	9	Unsteady gait	174	p.Arg208*	p.Tyr449Asn
**Mean ± SD**		**6,9 ± 0,3**	**9,6 ± 0,7**		**53,7 ± 21,7**		

^a^ These patients might even be considered as affected by the rare infantile CLN2 form.^7^

**FIGURE 1:**
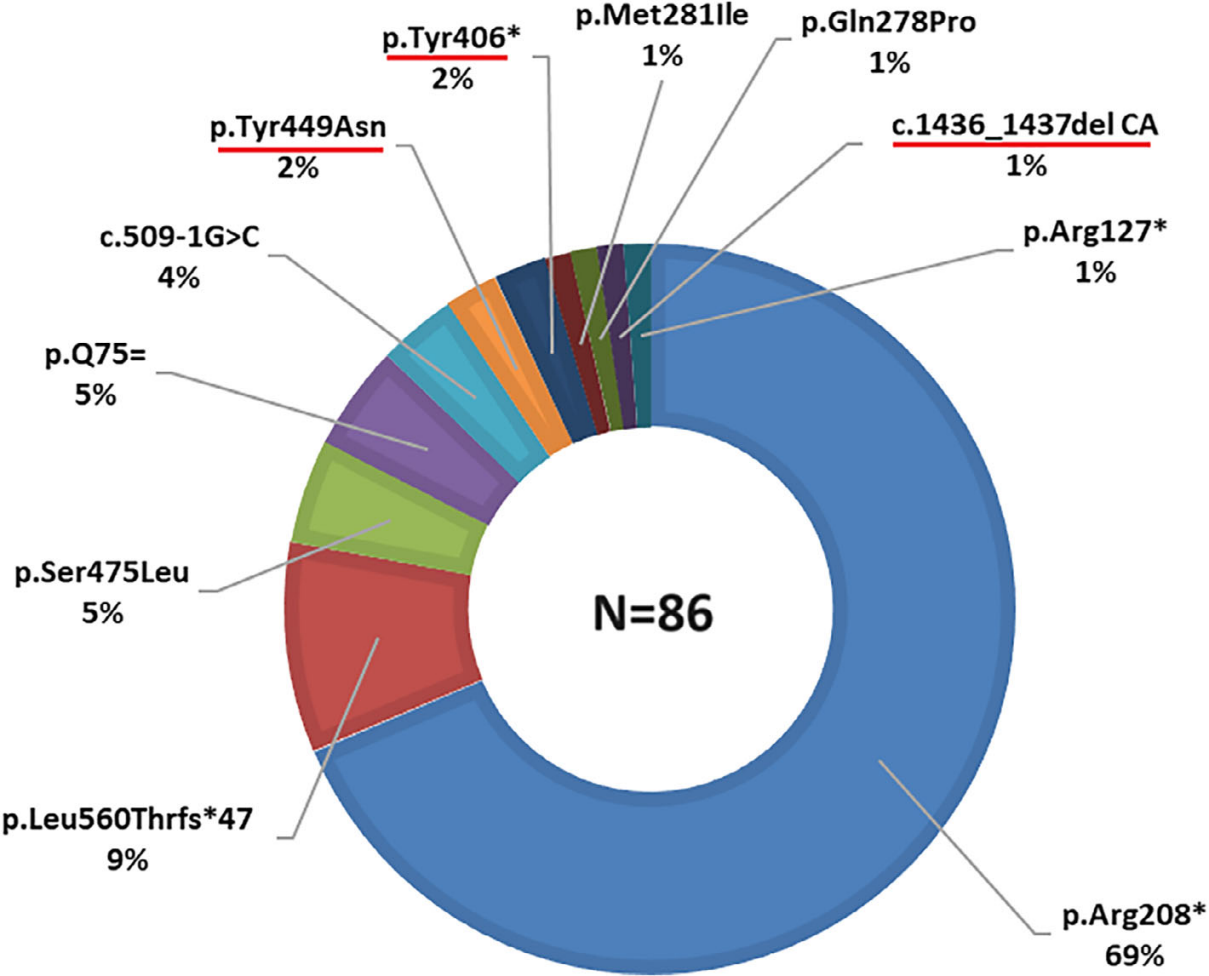
Pathogenic alleles in the *TPP1* gene were identified in Ukrainian patients with CLN2. The new pathogenic variants are underlined in red.

There were 11 genotypes among the CLN2 patients analyzed; of these, 8 were known worldwide,^7^ and three were novel (Figure 1). The novel variants of the *TPP1* gene were: a missense mutation p.Tyr449Asn in exon 11, a nonsense mutation p.Tyr406* in exon 10 and a deletion c.1436_1437del CA in exon 12. Cosegregation studies among direct family members were also performed to confirm that the variants were disease‐causing mutations.

The p.Arg208*variant, which is frequently reported in Europe, occurred in at least one allele in 38/43 families (88%) and accounted for 59/86 (69%) of disease‐associated alleles.

The p.Leu560Thrfs*47 and c.509‐1G > С variants were reported in eight and three unrelated patients, respectively, either in homozygous level (patient 4.1) or in compounds with the predominant mutated alleles. The allele p.Ser475Leu occurred in a homozygous state in two unrelated but geographically close patients from the northwest region of Ukraine, suggesting a likely founder effect. These four disease‐associated variants occurred in at least in one allele in 100% of Ukrainian families with CLN2. Other variants were private to single families, but the novel nonsense allele p.Tyr406* was found in a compound heterozygous state with p.Arg208* in two unrelated families.

All patients diagnosed with CLN2 in Ukraine had TPP1 deficiency in leukocytes ranging from 0 to 174 nmol/h/mg protein, compared to the control range of 179–681 nmol/h/mg protein. 41 patients (85%) presented with the classical late infantile type of disease (LI‐CLN2) with a mean age of manifestation (seizure) of 3,0 ± 0,1 years and a mean age of diagnosis of 4,1 ± 0,2 years. Patient 8.1, in whom onset was at approximately 1 year of age, could be classified as being affected by the rare infantile CLN2 (RI‐CLN2) form^7^ (Table 1). Mean TPP1 activity in this group of patients was 22,1 ± 3,7 (0–88) nmol/h/mg protein. 7 patients (15%) presented the late‐onset form of the disease (LO‐CLN2) with a mean age of manifestation of 6,9 ± 0,3 years (seizure) and a mean age of diagnosis of 9,6 ± 0,7 years. Mean TPP1 activity in this group of patients was 53,7 ± 21,7 (0–174) nmol/h/mg protein. In the vast majority of patients with LI‐CLN2 (94%) and in patient with RI‐CLN2, the disease's first manifestation was seizure. The remaining two patients with LI‐CLN2 first presented with an unsteady gait. In patients with LO‐CLN2, the disease's first manifestation had a different pattern: 29% experienced seizures, 42% unsteady gait, and 29% speech delay. It must be said that we did not find significant differences in the TPP1 activity in LI‐CLN2 patients and LO‐CLN2 patients (*p* < 0,05). In both clinical groups, both types of pathogenic variants, null and residual, were presented (Table 2).

**TABLE 2:** The number of affected patients in different clinical groups with a combination of null and residual pathogenic variants in the *TPP1* gene.

Combination of variant types	Genotypes	Number of patients with classical late infantile CLN2	Number of patients with juvenile CLN2
Null/null	p.Arg208*/p.Arg208* p.Arg208*/p.Leu560Thrfs*47 p.Leu560Thrfs*47/p.Leu560Thrfs*47 p.Arg208*/c.509‐1G > С p.Arg208*/p.Tyr406* p.Arg208*/p.Arg127* p.Arg208*/c.1436_1437del CA p.Arg208*/p.Met281Ile p.Arg208*/p.Gln75= p.Gln75=/p.Gln75=	38	5
Null/residual	p.Arg208*/p.Gln278Pro p.Arg208*/p.Tyr449Asn	1	2
Residual/residual	p.Ser475Leu/p.Ser475Leu	2	0

Thus, the data we obtained on the clinical and genetic features of CLN2 in patients from Ukraine supplements existing information on genotype–phenotypic correlations in this rare disease, which, in turn, lays the foundation for a personalized approach to the management of this disease.

## 4 DISCUSSION

Diagnosis of CLN2 disease is made through a complex of clinical findings, TPP1 enzyme deficiency, and/or molecular findings in TPP1.^2^ Historically, diagnoses of CLN subtypes have relied on histopathological techniques, such as an electron microscope evaluation of autofluorescent storage material morphology, together with a clinical review of disease onset and symptoms.^8^ Assaying of white blood cell TPP1 activity is now the mainstay of diagnosis for TPP1‐related diseases^2^ and is carried out when there is a suspicion of CLN2 or other CLN. Alongside the demonstration of deficient TPP1 enzyme activity, the detection of two pathogenic mutations in trans is considered the gold standard for CLN2 disease diagnosis.^2^ In addition, if a second variant is not identified, but enzyme activity is deficient, this can be used as evidence to classify any other potentially deleterious variants in the patient as well as provide a laboratory‐based diagnosis of CLN2 disease.^2^

With the knowledge of a shared molecular etiology, rather than being distinct entities, these diseases can be considered part of the same phenotypic spectrum that includes classic late‐infantile CLN2 disease and forms of atypical CLN2 disease.^8^ The majority of our patients (85%) had classic late‐infantile CLN2, one of them (patient 8.1) whose onset was approximately 1 year of age could be described as affected by the rare infantile CLN2 form, and 7 patients (15%) had the late‐onset form of the disease.^7^

Most of known variants identified in Ukrainian patients are associated with late infantile CLN2: out of 11 variants, three were nonsense (p.Arg208*, p.Arg127*, and p.Tyr406*), two were frameshift (p.Leu560Thrfs*47 and c.1436_1437del CA), two were splicing (c.509‐1G > С and p.Gln75=), and one was missense variant (p.Met281Ile), which is localized in highly conservative locus of the *TPP1* gene (https://www.ucl.ac.uk/ncl-disease/). This causes these variants to be classified as null mutations. Only two minor variants (p.Ser475Leu and p.Gln278Pro) with a summary frequency of 6% are likely to lead to residual TPP1 activity. The novel variants are nonsense mutation, deletion with frameshift, and missense variant in the highly conservative locus of the gene, which most likely also represents the null mutation. This genetic data could explain the high prevalence of the late infantile type of CLN2 among our patients. However, the lack of differences in the range of pathogenic variants in the *TPP1* gene in both clinical groups of patients should be noted (Table 2). This is consistent with no significant differences in the TPP1 activity in LI‐CLN2 patients and LO‐CLN2 patients, which may indicate that there are no etiological prerequisites for dividing patients into these clinical groups. Differentiation of the clinical groups is only possible through a review of the clinical data, with the most significant finding being the age of onset. The timing and nature of the disease's manifestation could be influenced by several factors, including the availability of complex alleles, the influence of adjacent or modifying genes, and the impact of environmental factors, epigenetics, etc.^14^ These factors impact the possibilities of the clinical prognosis and require additional studies.

It is worth noting that no significant features in the regional distribution of *TPP1* alleles were found – both the total number of patients and the frequencies of detected alleles are approximately evenly represented in all regions of Ukraine.

**FIGURE 2:**
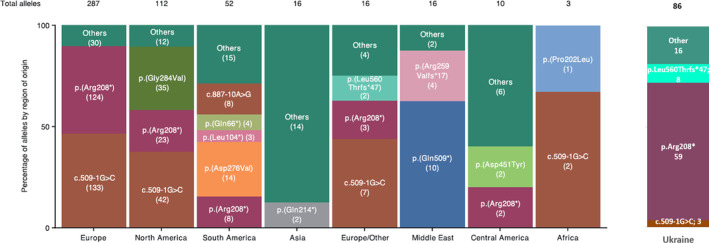
A comparison of the spectrum of a frequency of pathogenic variants in the TPP1 gene in Ukrainian CLN2 patients and patients from another region of origin. Adapted from Gardner et al.^3^

A comparison of the frequency of pathogenic variants in the *TPP1* gene, identified in Ukrainian CLN2 patients, with published data, demonstrates the significant differences (Figure 2).

The frequency of variant p.Arg208* in Ukrainian families is higher than in any other ethnic group, while variant c.509‐1G > С, typically found in European, South American, and African patients, is not so widespread in the Ukraine.

This finding is very important in the era of personalized medicine for developing population‐specific approaches to diagnosing CLN2, correctly interpreting the results of genetic analyses and determining therapeutic strategies for patients. This is of particular importance due to the existence of pathogenetic CLN2 enzyme replacement therapy, the effectiveness of which significantly depends on the early and accurate diagnosis of this disease.

Analysis of the molecular and genetic nature of a disease in a specific patient is mandatory as it not only confirms diagnosis but can help predict the clinical course of the disease and predict response to specific therapy.

## 5 CONCLUSIONS

Timely diagnosis facilitates the early initiation of appropriate disease‐specific care and enables families to make informed treatment decisions.^15^

The uniform and timely reporting of all variants not only benefits families by providing a definitive diagnosis but also allows genotype–phenotype correlations to be confirmed or reassessed. Comprehensive reporting and data sharing are essential as molecular genetic testing increases as a first‐line diagnostic test for pediatric‐onset neurological disease. To simplify the future genetic analysis of TPP1‐deficient patients in Ukraine, one might consider ruling out testing for the exon 6 (p.Arg208*), exon 13 (p.Leu560Thrfs*47), and intron 5 (c.509‐1G > С) variants and for patients from north‐west region of the Ukraine testing also for exon 11 (p.Ser475Leu), before starting costly complete TPP1/CLN2 screening. At present, these four most common Ukrainian mutations comprise 81% of the detected pathological alleles in Ukraine and appear in at least one allele in all affected Ukrainian families.

In most patients here described, genotype and phenotype correlation is in keeping with the data of previous studies. The clinical signs of the disease in patients with new, previously undescribed variants allowed us to augment existing data about genotype–phenotype correlations for CLN2 disease.

The combination of genotype and clinical form of the disease, identified in our patients, demonstrated that predicting the type and clinical course of the disease based on genotype is very complicated. The correlation of the type of genetic variant with the clinical manifestations of the disease in the patient makes it possible to use this data in the management of CLN2 and individually plan the most rational and effective management tactics.

## AUTHOR CONTRIBUTIONS

*Conceptualization and planning*: Nataliia Olkhovych and Nataliia Gorovenko. *Methodology*, *validation*: Nataliia Olkhovych and Nataliia Mytsyk. *Formal analysis*, *writing*—*original draft preparation*: Nataliia Olkhovych, Nataliia Mytsyk. *Data curation*: Nataliia Pichkur, Nataliia Samonenko, Tetiana Shklyarskaya. *Resources*, *supervision*, *and project administration*: Nataliia Olkhovych. *Investigation*, *writing*—*review*: Rodolfo Tonin, Svitlana Kormoz, Iryna Hregul, Volodymyr Olkhovych, Olexandr Buryak. *Proofreading the manuscript and editing*: Amelia Morrone. All authors have read and agreed to the version of the manuscript.

## FUNDING INFORMATION

This research did not receive any specific grant from funding agencies in the public, commercial, or not‐for‐profit sectors. The authors confirm independence from the sponsors, the content of the article has not been influenced by the sponsors.

## CONFLICT OF INTEREST STATEMENT

Nataliia Olkhovych, Nataliia Pichkur, Rodolfo Tonin, Nataliia Mytsyk, Svitlana Kormoz, Iryna Hregul, Nataliia Samonenko, Tetiana Shklyarskaya, Volodymyr Olkhovych, Olexandr Buryak, Amelia Morrone, and Nataliia Gorovenko declare that they have no conflict of interest.

## ETHICS STATEMENT

All procedures followed were in accordance with the ethical standards of the responsible committee on human experimentation (institutional and national) and with the Helsinki Declaration of 1975, as revised in 2000 (5). Informed consent was obtained from all patients for being included in the study. Proof that informed consent was obtained must be available upon request.
